# Physician experiences and preferences in the treatment of HR+/HER2− metastatic breast cancer in the United States: a physician survey

**DOI:** 10.1002/cam4.580

**Published:** 2015-12-21

**Authors:** Peggy L. Lin, Yanni Hao, Jipan Xie, Nanxin Li, Yichen Zhong, Zhou Zhou, James E. Signorovitch, Eric Q. Wu

**Affiliations:** ^1^ Analysis Group, Inc.BostonMassachusetts; ^2^ Novartis Pharmaceuticals CorporationEast HanoverNew Jersey; ^3^ Analysis Group, Inc.New YorkNew York

**Keywords:** Chemotherapy, endocrine therapy, everolimus, fulvestrant, metastatic breast cancer, survey

## Abstract

Sequential endocrine therapy (ET) is recommended for postmenopausal women with hormone receptor‐positive (HR+)/human epidermal growth factor receptor 2‐negative (HER2−) metastatic breast cancer (mBC) and without visceral symptoms. Chemotherapy (CT) can be considered after sequential ETs, but is associated with adverse side effects. We assessed physicians' preferences and self‐reported prescribing patterns for ET and CT in the treatment of HR+/HER2− mBC at community practices in the United States. Community‐based oncologists/hematologists from a nationwide online panel who treated postmenopausal women with HR+/HER2− mBC were invited to complete a survey, blinded to the identity of study sponsor. Treatment preferences were collected by treatment class of ET‐based regimens versus CT and by agent for postmenopausal HR+/HER2− mBC patients after prior nonsteroidal aromatase inhibitor use in the adjuvant or mBC setting. Among 213 physicians who completed the survey, 78% were male, 71% were based in small/intermediate practices (2–9 oncologists/subspecialists), 55% had >10 years of experience, and 58% referred to the National Comprehensive Cancer Network Guidelines when treating mBC. Among first‐line ETs, anastrozole was the most frequently used treatment (35%), followed by everolimus‐based (EVE, 34%) and fulvestrant‐based (FUL, 15%) therapy. After first‐line ET, the most preferred second‐ and third‐line treatments were ET monotherapy (48% and 39%), ET combination therapy (31% and 19%), and CT monotherapy (13% and 30%). Comparing EVE versus FUL, physicians preferred EVE in all lines but first line. Efficacy was the most important consideration for treatment choice. Physicians prescribed CT in early lines mainly because of visceral symptoms. This survey of treatment patterns for HR+/HER2− mBC in community practice suggested that after first‐line ET, ET mono‐ or combination therapy was commonly used for the second‐ and third‐line treatments and CT monotherapy for third‐ or later line treatments. CTs were used in early lines for patients with visceral symptoms.

## Introduction

Breast cancer (BC) is the most common cancer among women 1, 2. It is also the second most common cause of cancer‐related deaths in women globally 2, and the third leading cause of cancer‐related death in the United States 1. Metastatic breast cancer (mBC) occurs in up to 5% of the new diagnoses 1 and it develops in nearly 30–40% of recurrent BCs 3, 4. Survival for mBC is poor: the median survival time is 2–3 years 5, 6, 7, 8, 9 and only one in four patients are still alive at 5 years postdiagnosis of metastatic disease 1, 10.

Existing National Comprehensive Cancer Network (NCCN) treatment guidelines recommend sequential endocrine therapy (ET) for postmenopausal women with hormone receptor‐positive (HR+)/human epidermal growth factor receptor 2‐negative (HER2−) mBC and without visceral symptoms 11. Chemotherapy (CT) can be considered after sequential endocrine therapies—or in visceral or rapidly advancing disease 11,—but it is generally associated with various serious side effects 12, 13. American Society of Clinical Oncology (ASCO) treatment guidelines for HR+/HER2− mBC likewise recommend the use of ET as first‐line treatment in postmenopausal women, while also including CT in the list of possible first‐line treatments for mBC 14. In the latter case, ASCO guidelines note that single agent CT is preferable to combination CT due to potentially higher toxicity with multiple agents 14, with the exception of fast‐acting disease, where immediate response to medication is prioritized over limiting treatment toxicity 15, 16, 17, 18.

Both objective factors, such as treatment guidelines, and subjective factors, such as physician preferences, have been shown to influence physicians' disease management recommendations and treatment patterns for their BC patients 19, 20, 21, 22, 23, 24. While several studies have focused on physician variability in treating early BCs 19, 21, 23, 25, 26, 27, 28, 29, there is limited information on physician treatment preferences for advanced BC 30 and their real‐world prescribing experiences 31. The objective of this physician survey is to describe physicians' preferences and self‐reported prescribing patterns in treating HR+/HER2− mBC among community oncology practices in the United States.

## Methods

### Data source

Community‐based oncologists/hematologists who treated postmenopausal women with HR+/HER2− stage IV mBC were recruited from an online nationwide panel of over 9500 medical oncologists/hematologists and were invited to complete a survey online. The study sponsor and authors did not participate or influence the process of physician recruitment. At the time of the survey (Q2 2014), the physicians did not know the identity of the study sponsor, and their identity was blinded to the study sponsor and all coauthors. The participating physicians were compensated for the time spent completing the survey. Along with the survey, participating physicians extracted chart information on eligible patients, and results of the chart review are reported separately 32. Briefly, patient chart and physician survey information was recorded in an electronic case report form (eCRF), accessible to the participating physicians through a secure online portal. Prior to launch, the eCRF had been extensively tested for programming logic and consistency; additionally, three physicians completed the questionnaire during a pilot run, in order to verify the clarity and understandability of the questionnaire.

Physician‐recalled treatment preferences and prescribing patterns, categorized by treatment class—ET versus CT—and by specific agent used were collected for postmenopausal HR+/HER2− mBC patients who had BC recurrence or progression on or after a nonsteroidal aromatase inhibitor (AI) treatment in the adjuvant or mBC setting and who started a new treatment for mBC between 1 July 2012 and 15 April 2013. In addition, the survey also collected information on physician characteristics such as gender and years of practice. Data were deidentified at the time of collection and comply with the patient confidentiality requirements of the Health Insurance Portability and Accountability Act (HIPAA). The study was approved by the New England Institutional Review Board.

### Study measures

#### Physician characteristics and physician‐reported characteristics of postmenopausal HR+/HER2− mBC patients

Physician characteristics examined in the current study were gender, practice size, region, and years of practice experience.

Based on their experience, the surveyed physicians reported on several characteristics of their patients (as a collective group, not based on individual patient charts) who would meet the eligibility criteria described above (i.e., postmenopausal HR+/HER2− mBC patients who had BC recurrence or progression on or after a nonsteroidal AI treatment in the adjuvant or mBC setting). These characteristics included estimated number and percentage of patients treated, categorized by treatment class and by line of treatment, typical duration (in months) of first‐line treatment, typical survival time from initiation of first‐line treatment, and typical number of lines of endocrine therapies that patients received before CT initiation.

#### Prescribing patterns

All physicians were asked to rank a list of known guidelines or clinical pathways for the treatment of mBC in the order of importance, based on their usage preferences.

Physicians' therapeutic preferences for the treatment of their HR+/HER2− mBC patients were collected by asking physicians about their preferred therapy by treatment class (ET vs. CT), treatment regimen (individual agents, monotherapy vs. combination therapy), line of therapy, and by early (first or second line) versus later lines. Aside from general category choice, physician preference for everolimus (Afinitor, Novartis Pharmaceuticals Corporation, East Hanover, NJ) or fulvestrant (FUL) (Faslodex, AstraZeneca Pharmaceuticals LP, Wilmington, DE) therapies was investigated. In the current study, everolimus‐based therapy (including everolimus monotherapy, combination therapy of everolimus and ET, or combination therapy of everolimus and CT) was grouped in the same treatment class as ET when assessing physician preferences by class of therapy (endocrine vs. CT), and reported separately when assessing physician preferences and usage of everolimus‐based therapy. Physician preferences for everolimus‐based therapy and FUL‐based therapy (including FUL monotherapy or combination therapy of FUL with other ET) were collected by line of treatment.

#### Reasons for treatment choices

For physicians who reported having prescribed a specific treatment (e.g., ET, CT) to their patients that would meet the eligibility criteria of the current study, reasons for treatment choices were collected for prescribing that treatment in general (i.e., nonspecific to individual patients), stratified by line of treatment. The following information was collected on the reasons underlying physicians' treatment decisions: (1) reasons for prescribing a certain treatment, stratified by treatment class and by line of therapy, (2) factors influencing the choice of treatment regimens, (3) reasons for discontinuation, and (4) among physicians who reported having prescribed CT, the point in the patient's treatment course when the physician typically initiated CT (e.g., after patient experienced treatment failure with a specific line of therapy).

### Statistical analysis

Summary statistics were used to describe physician characteristics and physician‐estimated patient characteristics. Medians and ranges were reported for continuous variables. Frequencies and percentages were reported for categorical variables.

For physician prescribing patterns and reasons for treatment choices, the following information was summarized: (1) the proportion of physicians selecting specific guidelines/clinical pathways (ranked as first, second, or third choice in terms of importance) that they referred to when treating mBC, (2) the proportion of physicians preferring specific treatments, stratified by class (ET vs. CT) and by individual therapeutic agent, and (3) the proportion of physicians selecting different reasons for treatment choices and reasons for discontinuation, stratified by treatment class and by line.

## Results

### Physician characteristics and physician‐reported characteristics of postmenopausal HR+/HER2− mBC patients

Among the 213 physicians who completed the survey, 78% were male, 71% worked in a small/intermediate practice setting with 2–9 oncologist/subspecialists, and over half (55%) had more than 10 years of practice experience (Table 1). There was a good representation of the different regions of the country among the physicians who completed the survey, although a slightly higher proportion of physicians were from the West (42%) than from any other region of the United States (Table 1).

**Table 1:** Physician characteristics

	All physicians (*N* = 213)
Demographic characteristics, *n* (%)	
Male	166 (77.9)
Female	47 (22.1)
Practice characteristics, *n* (%)	
Size of primary practice setting	
Individual practice (1 oncologist)	23 (10.8)
Small/intermediate (2–9 oncologists/specialists)	152 (71.4)
Large (10 oncologists/specialists or more)	38 (17.8)
Region of primary specialty setting in the United States	
Northeast	41 (19.2)
Midwest	24 (11.3)
South	58 (27.2)
West	90 (42.3)
Years of practice experience	
Less than 5 years	16 (7.5)
5–10 years	80 (37.6)
More than 10 years	117 (54.9)
Physician estimates of their mBC patients' treatment characteristics and outcomes	
Treatment duration (months) of first‐line therapy for mBC	
Mean (SD)	10.7 (4.9)
Median (range)	10.0 (2.0, 25.0)
Typical survival time (months) from initiation of first‐line therapy for mBC	
Mean (SD)	25.0 (17.0)
Median (range)	24.0 (2.0, 100.0)
Number of lines of endocrine therapies prior to chemotherapy initiation	
Mean (SD)	2.5 (0.9)
Median (range)	3.0 (0.0, 6.0)

mBC, metastatic breast cancer.

The surveyed physicians had treated, on average, 22 patients (per practitioner) that would meet the eligibility criteria of the current study. Among these patients, the median treatment duration of first‐line therapy, based on physician recall, was 10 months (range: 2–25 months). The mean survival time, measured from the initiation of first‐line therapy for mBC, was estimated to be 24 months (range: 2–100 months). Based on physician recall, patients typically received three lines of ET (range: 0–6 lines) prior to CT initiation (Table 1).

### Prescribing patterns**—**overall (ET and CT)

**Figure 1:**
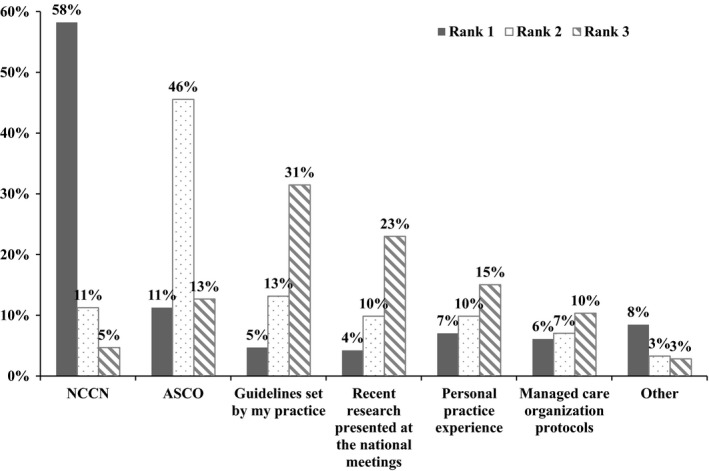
Guideline or clinical pathway preferences for the treatment of mBC (213 survey respondents).

Among the 213 physicians who completed the survey, 58% of physicians (*N* = 124) selected the NCCN guidelines 11 as the most important guidelines that they referred to when treating mBC (Fig. 1). Other important guidelines, listed in descending order of importance, included ASCO 33, guidelines set by the practice, and recent research presented at national meetings.

**Figure 2:**
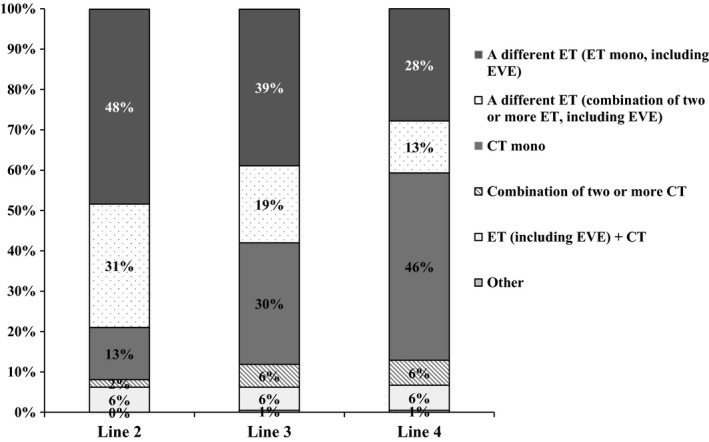
Physician‐reported prescribing patterns after first‐line endocrine therapy (209 survey respondents).

After first‐line ET, the most preferred treatments reported by physicians for second‐line therapy included a different endocrine monotherapy (48%, 101 of 209 physicians), a different endocrine combination therapy (31%, 64/209), and CT monotherapy (13%, 27/209) (Fig. 2). For third‐line treatment, the most preferred treatments were a different endocrine monotherapy (39%, 81 of 209 physicians), a different endocrine combination therapy (19%, 40/209), and CT monotherapy (30%, 63/209). For fourth‐line treatment after first‐line ET, the most preferred treatments were a different endocrine monotherapy (28%, 58 of 209 physicians), a different endocrine combination therapy (13%, 27/209), and CT monotherapy (46%, 97/209).

For patients who had received early lines of CT (as first‐ or second‐line treatments), the most preferred subsequent therapies were a different CT (including monotherapy and combination CT, 64%, 129 of 201 physicians) and everolimus‐based ET (19%, 39/201).

#### Prescribing patterns*—*ET

**Figure 3:**
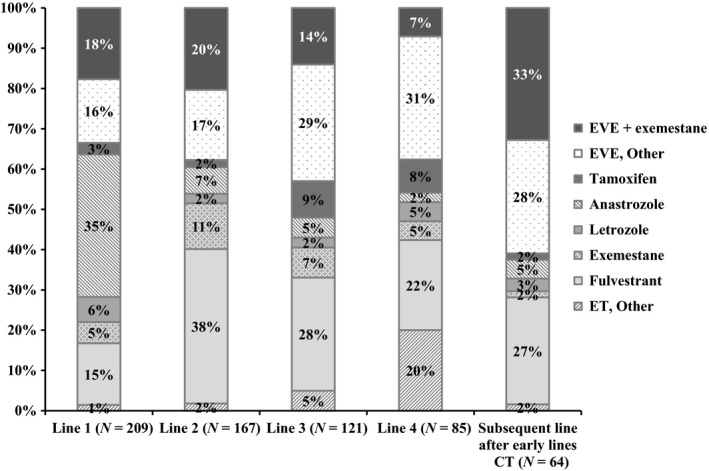
Physician‐reported preferences for endocrine therapy.

A total of 209 physicians responded to survey questions relating to first‐line endocrine therapies. Based on physician recall, anastrozole was the most frequently used treatment (35%, 74 of 209 physicians) among first‐line endocrine therapies, followed by 34% (60/209) choosing everolimus‐based therapy, and 15% (32/209) choosing FUL‐based therapy (Fig. 3).

Everolimus‐based and FUL‐based therapies were also among the top endocrine therapies preferred by physicians for the second‐, third‐, fourth‐line (after first‐line ET) treatments and as subsequent line of treatment after early lines of CT. For second‐line treatment, 37% (*N* = 63) of the 167 surveyed physicians indicated a preference for everolimus‐based therapy and 38% (*N* = 64) for FUL‐based therapy. Everolimus‐based therapy was the preferred ET in third and later lines. For third‐line treatment, 43% (*N* = 52) of the 121 physicians indicated a preference for everolimus‐based and 28% (*N* = 34) for FUL‐based therapies. For fourth‐line and subsequent treatments, 38% (*N* = 32) of the 85 surveyed physicians indicated a preference for everolimus‐based and 22% (*N* = 19) for FUL‐based therapies. Finally, for the subsequent line of treatment after the early lines of CT, 61% (*N* = 39) of the 64 physicians indicated a preference for everolimus‐based and 27% (*N* = 17) for FUL‐based therapies (Fig. 3).

Among the 205 physicians who reported their preferences between everolimus‐based and FUL‐based therapies, more preferred everolimus‐based therapy in second‐, third‐, and fourth line of treatments over FUL‐based therapy, although the percentages were numerically close for second line and statistical testing was not conducted to determine if the differences were statistically significant (Table 2). In addition, a high proportion of physicians indicated equal preference for third‐line (48%, 98 of 205 physicians), and no preference for either treatment for fourth‐line (44%, 90/205) treatments.

**Table 2:** Physician‐reported^a^ preferences between everolimus‐ and fulvestrant‐based therapies by line of treatment

Preference for everolimus‐based vs. fulvestrant‐based therapies in each line in the stage IV metastatic setting, *n* (%)	First line	Second line	Third line	Fourth line
Prefer everolimus	66 (32.2)	82 (40.0)	52 (25.4)	38 (18.5)
Prefer fulvestrant	79 (38.5)	77 (37.6)	35 (17.1)	18 (8.8)
Equal preference	27 (13.2)	39 (19.0)	98 (47.8)	59 (28.8)
Neither	33 (16.1)	7 (3.4)	20 (9.8)	90 (43.9)

a A total of 205 physicians responded to this question.

#### Prescribing patterns—CT

**Figure 4:**
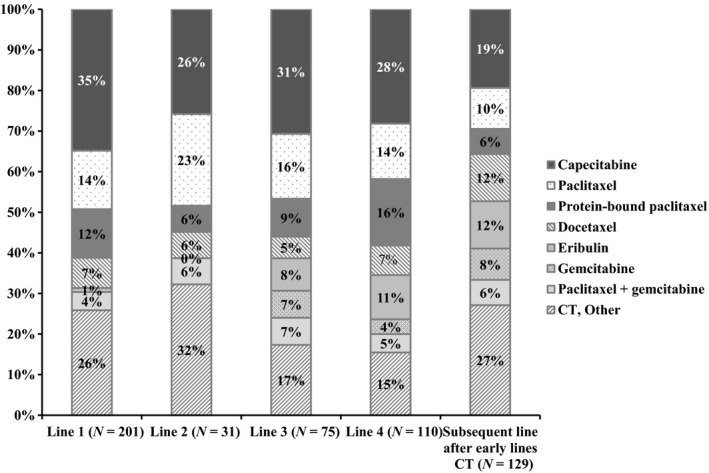
Physician‐reported preferences for chemotherapy.

Among CT treatments, capecitabine was the most frequently used agent across different lines of therapy, followed by paclitaxel/protein‐bound paclitaxel (Fig. 4). Of the physicians who reported CT preferences, physicians who chose capecitabine accounted for 35% (70 of 201 physicians) for first line, 26% (8/31) for second line, 31% (23/75) for third line, 28% (31/110) for fourth line, and 19% (25/129) for subsequent lines of treatments after early lines of CT. Paclitaxel or protein‐bound paclitaxel usage reported by the physicians was 26% (24 of 201 physicians) for first line, 29% (2/31) for second line, 25% (7/75) for third line, 30% (18/110) for fourth line, and 16% (8/129) for subsequent lines of treatments after early lines of CT (Fig. 4).

### Reasons for treatment choices—overall (ET and CT)

Among the physicians who responded to this question, efficacy was the most important factor considered by physicians when prescribing ET (including everolimus‐based therapy) or CT. For first‐line treatment, efficacy was overwhelmingly the most important consideration for the treatment choice (68%, 144 of 213 physicians) (Table 3). For second‐line treatment after first‐line ET, the most important factors were efficacy (51%, 106 of 209 physicians), followed by new mechanism of action (MOA) (19%, 39/209), and tolerability (14%, 30/209). For subsequent therapy after early lines of CT, the most important factors considered were efficacy (54%, 108 of 201 physicians), followed by tolerability (14%, 29/201), and new MOA (14%, 28/201). Correspondingly, lack of efficacy was the most important reason for discontinuing the second‐line treatment after the first‐line ET. Other reasons for treatment discontinuation were disease progression with imaging evidence (85%, 177 of 209 physicians), disease progression without imaging evidence (8%, 17/209), and nonresponse to treatment, without progression (4%, 9/209).

**Table 3:** Reasons for treatment choices or discontinuation—overall (endocrine therapy and chemotherapy)

	All physicians (*N* = 213)
What is the most important factor that you normally consider when prescribing the first‐line therapy in the stage IV metastatic setting?^a^, *n* (%)	
Efficacy of the treatment	144 (67.6)
Treatment guidelines	20 (9.4)
New mechanism of action of the treatment	3 (1.4)
Tolerability of treatment	16 (7.5)
Prior exposure and/or response to adjuvant endocrine therapy (if applicable)	3 (1.4)
Tumor and disease burden (e.g., extent of visceral metastasis, sign of visceral crisis)	18 (8.5)
Patient age, comorbidity, and performance status	1 (0.5)
Patient current overall quality of life (QoL) and impact of treatment on QoL	8 (3.8)
What is the most important factor that influences your choice of the second‐line therapy after first‐line endocrine therapy (including everolimus) in the stage IV metastatic setting?^a^, *n* (%)	
Number of physicians who responded	209 (98.1)
Efficacy of the treatment	106 (50.7)
New mechanism of action of the treatment	39 (18.7)
Tolerability of the treatment	30 (14.4)
The type of the first‐line endocrine therapy agent and/or the response to the first‐line endocrine therapy agent	15 (7.2)
Tumor and disease burden (e.g., tumor size, metastatic sites, extent of visceral metastasis, sign of visceral crisis)	9 (4.3)
Patient age, comorbidity, and performance status	2 (1.0)
Patient current overall QoL and impact of treatment on QoL	8 (3.8)
What is the most important factor that influences your choice of the subsequent therapy after the early lines of chemotherapy (first or second line) in the stage IV metastatic setting?^a^, *n* (%)	
Number of physicians who responded	201 (94.4)
Efficacy of the treatment	108 (53.7)
New mechanism of action of the treatment	28 (13.9)
Tolerability of the treatment	29 (14.4)
Patient's tolerability to further lines of chemotherapy	7 (3.5)
Tumor and disease burden (e.g., tumor size, metastatic sites, extent of visceral metastasis, signs of visceral crisis)	17 (8.5)
Patient age, comorbidity, and performance status	1 (0.5)
Patient current overall QoL and impact of treatment on QoL	7 (3.5)
Treatment route, dosing frequency, patient adherence	2 (1.0)
Cost of drug, patient copay, insurance, and access	2 (1.0)
What is the most common reason for discontinuing the second‐line therapy initiated after the first‐line endocrine therapy (including everolimus) in a stage IV metastatic setting?^a^, *n* (%)	
Number of physicians who responded	209 (98.1)
Disease progression (with imaging evidence)	177 (84.7)
Disease progression (without imaging evidence)	17 (8.1)
Nonresponse without progression	9 (4.3)
Drug toxicity/drug intolerance	4 (1.9)
Other nonmedical reason^b^	1 (0.5)
Death	1 (0.5)

a The proportion of each factor or reason is measured among physicians who responded.

b Other nonmedical reason: disease stabilization.

#### Reasons for treatment choices—ET

When asked about the most important reasons for prescribing ET as the first‐line therapy for mBC, the majority of physicians selected good efficacy (48%, 100 of 209 physicians) and guideline recommendation (38%, 80/209) as the top reasons (Table 4). In terms of the most important factors that influenced the physician's choice of the first‐line ET, the most important factors considered were efficacy (58%, 121 of 209 physicians), followed by new MOA (16%, 33/209), and tolerability (12%, 24/209). The most commonly reported reasons for discontinuing first‐line ET were disease progression with imaging evidence (79%, 164 of 209 physicians), disease progression without imaging evidence (12%, 24/209), and nonresponse without progression (7%, 15/209).

**Table 4:** Reasons for treatment choices or discontinuation—endocrine therapy (including everolimus‐based therapy)

	All physicians (*N* = 213)
What is the most important reason for prescribing endocrine therapy (including everolimus) as the first‐line therapy in the stage IV metastatic setting?^a^, *n* (%)	
Number of physicians who responded	209 (98.1)
It is recommended by the guidelines	80 (38.3)
Endocrine therapies have good efficacy in this population	100 (47.8)
Endocrine therapies are safe to use	13 (6.2)
Prior exposure and/or response to adjuvant endocrine therapy (if applicable)	6 (2.9)
Patients cannot tolerate chemotherapies	6 (2.9)
Endocrine therapies are convenient to use	3 (1.4)
Endocrine therapies are affordable	1 (0.5)
What is the most important factor that influences your choice of the first‐line endocrine therapy (including everolimus) for stage IV mBC among the following patients?^a^ *n* (%)	
Number of physicians who responded	209 (98.1)
Efficacy of an endocrine therapy treatment	121 (57.9)
New mechanism of action of an endocrine therapy treatment	33 (15.8)
Tolerability of an endocrine therapy treatment	24 (11.5)
Prior exposure and/or response to adjuvant endocrine therapy (if applicable)	14 (6.7)
Tumor and disease burden (e.g., tumor size, metastatic sites, extent of visceral metastasis, signs of visceral crisis)	9 (4.3)
Patient age, comorbidity, and performance status	1 (0.5)
Patient current overall QoL and impact of treatment on QoL	5 (2.4)
Treatment route, dosing frequency, patient adherence	1 (0.5)
Cost of drug, patient copay, insurance, and access	1 (0.5)
What is the most common reason for discontinuing the first‐line endocrine therapy (including everolimus) for stage IV mBC among the following patients?^a^, *n* (%)	
Number of physicians who responded	209 (98.1)
Disease progression (with imaging evidence)	164 (78.5)
Disease progression (without imaging evidence)	24 (11.5)
Nonresponse without progression	15 (7.2)
Drug toxicity/drug intolerance	3 (1.4)
Other nonmedical reason^b^	1 (0.5)
Death	2 (1.0)

mBC, metastatic breast cancer; QoL, quality of life.

a The proportion of each reason or factor is measured among physicians who responded.

b Other nonmedical reason: disease stabilization.

#### Reasons for treatment choices—CT

Among physicians who reported ever prescribing CT in the early lines, the most common reasons for doing so included the presence of visceral metastasis/crisis or symptomatic disease presentation (30%, 60/201 of 201 physicians), guidelines recommendation (27%, 55/201), good treatment efficacy (20%, 40/201), and fast disease progression (17%, 34/201) (Table 5). The most important factors that influenced physicians' choice of the early lines of CT included new MOA (55%, 110/201 of 201 physicians), nonsensitivity or nonresponse to prior adjuvant ET (15%, 30/201), and patient age, comorbidity, and performance status (15%, 30/201).

**Table 5:** Reasons for treatment choices or discontinuation—chemotherapy

	All physicians (*N* = 213)
What is the most important reason for prescribing chemotherapy as early lines of therapy, that is, the first or second line in the stage IV metastatic setting?^a^, *n* (%)	
Number of physicians who responded	201 (94.4)
It is recommended by the guidelines	55 (27.4)
Chemotherapies have good efficacy in this population	40 (19.9)
Patients experience fast progression	34 (16.9)
Patients have visceral metastasis/crisis or are symptomatic	60 (29.9)
Patient is not “hormone sensitive”, that is, nonresponse to adjuvant endocrine therapy	10 (5.0)
Patients can tolerate chemotherapies	2 (1.0)
At what point do you typically initiate chemotherapy for the following patients who have endocrine therapy (including everolimus) as the first‐line treatment in a stage IV metastatic setting?^a^, *n* (%)	
Number of physicians who responded	209 (98.1)
When patients experience treatment failure with first‐line endocrine therapy	41 (19.6)
When patients experience treatment failure with second‐line endocrine therapy	49 (23.4)
When patients experience treatment failure with third‐line endocrine therapy	50 (23.9)
When patients experience early relapse (within 6 months) while on a line of endocrine therapy	13 (6.2)
When patients experience early relapse (within 4 months) while on a line of endocrine therapy	11 (5.3)
When patients have organ‐threatening disease or visceral crisis	37 (17.7)
When patients have symptomatic disease	3 (1.4)
When disease progresses fast	5 (2.4)
What is the most important factor that influences your choice of the early lines (first or second line) of chemotherapy in the metastatic setting?^a^, *n* (%)	
Number of physicians who responded	201 (94.4)
New mechanism of action of the chemotherapy treatment	110 (54.7)
Tolerability of the chemotherapy treatment	15 (7.5)
Nonsensitivity or nonresponse to prior adjuvant endocrine therapy	30 (14.9)
Tumor and disease burden (e.g., tumor size, metastatic sites, extent of visceral metastasis, signs of visceral crisis)	5 (2.5)
Patient age, comorbidity, and performance status	30 (14.9)
Patient current overall quality of life (QoL) and impact of treatment on QoL	1 (0.5)
Treatment route, dosing frequency, patient adherence	6 (3.0)
Cost of drug, patient copay, insurance, and access	4 (2.0)
What is the most common reason for discontinuing early lines (first or second line) of chemotherapy in the stage IV metastatic setting?^a^, *n* (%)	
Number of physicians who responded	201 (94.4)
Disease progression (with imaging evidence)	166 (82.6)
Disease progression (without imaging evidence)	14 (7.0)
Nonresponse without progression	12 (6.0)
Drug toxicity/drug intolerance	7 (3.5)
Other nonmedical reason^b^	1 (0.5)
Death	1 (0.5)

a The proportion of each factor or reason is measured among physicians who responded.

b Other reason for discontinuing the early lines of chemotherapy: disease stabilization.

For mBC patients who had received ET for first line, physicians reported that they typically initiated CT when patients experienced treatment failure with first‐line ET (20%, 41 of 209 physicians), failure with second‐line ET (23%, 49/209), failure with third‐line ET (24%, 50/209), or when patients had organ‐threatening disease or visceral crisis (18%, 37/209).

The most important reasons for discontinuing early lines of CT were disease progression with or without imaging evidence (90%, 180 of 201 physicians) and nonresponse without progression (6%, 12/201).

## Discussion

In this study of physician preferences for the treatment of mBC, surveyed physicians indicated that the NCCN guidelines were the most frequently consulted guidelines when deciding therapy course. Consistent with this result, the surveyed physicians reported prescribing an average of three lines of ET to patients with stage IV mBC before initiating any course of CT. However, it is worth noting that for patients who experienced failure of the first‐line ET (did not experience tumor shrinkage in response to treatment or disease stabilization), a significant proportion of physicians reported that they would prescribe CT (including single agent CT, combination of two or more chemotherapies, or the combination of ET with CT) for the second (21%, 44 of 209 physicians) or third line of treatment (42%, 87 of 209 physicians). Visceral symptoms constituted an important reason for prescribing early lines of CT in the surveyed physicians' experience. This finding is also consistent with the recommendations of NCCN and ASCO guidelines, which support CT use in patients with visceral or fast‐progressing disease.

Everolimus‐based and FUL‐based therapies were the preferred endocrine therapies in the second, third, and fourth lines of treatment after first‐line ET or after early lines of CT. Comparing the two treatments by line, physicians reported greater preference for everolimus‐based therapies over FUL‐based therapies in all lines but the first line.

Across treatment lines, efficacy was the most important consideration for treatment choice. Tolerability, a new MOA, the type of prior therapy, and the patient's response to the previous treatment were also important influencing factors. For patients initiating CT as an early line of treatment, the choice of a specific regimen was based mainly on considerations of a new MOA, the patient's prior response to endocrine therapies, and patient age, comorbidity, and performance status. In terms of reasons for discontinuation, disease progression was the predominant reason for the physician's decision to discontinue a treatment, irrespective of treatment type or line.

To our knowledge, this is the first study to report on physician preferences in the treatment of mBC and physician‐reported reasons for therapy choice, aside from a European study discussing physician preferences on whether to continue or stop CT after induction with six cycles of therapy 30. That study found that although almost half of the physicians indicated a preference for continuous treatment for a 3‐month gain in time to progression, this action was not supported by clinical evidence, as no significant difference was found in progression‐free or overall survival in these patients 30. However, this study was conducted more than a decade ago and physician preferences might have evolved in the light of recent phase III trial findings 34. Another European study (surveyed during July 2008–June 2010) described treatment patterns as reported by physicians, but did not include physician preference in the survey 31. The current study addressed the literature gap on physicians' preferences and self‐reported prescribing patterns in the real‐world setting.

It must be noted that this study has several limitations. First, as with all studies of a descriptive, retrospective survey design, the current findings are subject to physician recall errors 35. Second, this study was based on physicians' experiences and general preferences and was not designed to take into account individual patient characteristics. Treatment preferences and physician‐recalled prescribing patterns were collected for postmenopausal HR+/HER2− mBC patients as a collective group who has failed prior nonsteroidal AI in adjuvant or mBC setting. No further differentiation was made between recurrent and de novo patients, or among recurrent patients, based on the adjuvant therapy used, and the time elapsed between the end of last adjuvant ET and the initiation of first‐line therapy for mBC. Future studies are needed to further address these potential influencing factors. The findings of this study should not be used to make treatment decisions or to specify drug selection. Finally, the study's sample size of responders, relative to the complete panel of physicians who were invited to participate, was small, possibly due to the eligibility criteria used—but such response rate was comparable to that of other online physician survey studies 36, 37. Yet the reported findings may not be completely representative of or generalizable to the larger community of oncologists that treat BC patients in the United States. Nonetheless, despite these limitations, retrospective survey studies remain a commonly used, convenient, and valuable source of real‐world information on clinical practice patterns 38 and, to our knowledge, the current study is the first to provide evidence on physicians' experiences and preferences of treatment patterns for HR+/HER2− mBC in community practices in the United States during the new era of targeted therapies.

## Conclusion

The treatment patterns reported by the physicians surveyed in this study were generally consistent with treatment guideline recommendations. For patients with HR+/HER2− mBC, physicians typically prescribed a median of three lines of ET prior to CT initiation. After first‐line ET, monotherapy or combination ET was commonly used in the second and third line, and CT monotherapy in the third or later lines of treatment. For patients with visceral symptoms, physicians were more likely to prescribe CT as early lines of treatment. After first‐line ET, everolimus‐based and FUL‐based therapies were both frequently used in the next line(s) of treatment, but the surveyed physicians reported a greater preference for everolimus‐based therapy in all subsequent treatment lines. Exemestane, anastrozole, and tamoxifen were other ET commonly used in the second and third lines of treatment, while capecitabine, paclitaxel, and protein‐bound paclitaxel were the most commonly used CTs in the third or later lines of treatment.

## Conflict of Interest

This study was funded by Novartis Pharmaceuticals Corporation. Y. H. is an employee of Novartis and owns stock/stock options. P. L. L., J. X., N. L., Y. Z., Z. Z., J. E. S., E. Q. W. are employees of Analysis Group, which has received consultancy fees from Novartis for this project. In addition, Analysis Group provides research and consulting services to a large number of for‐profit healthcare companies. In the past 3 years, P. L. L. has provided research/consulting support for AbbVie, AstraZeneca, Bayer, LifeScan, Shire, and Takeda projects; J. X. has provided research/consulting support for AbbVie, Astellas, AstraZeneca, Bayer, Ethicon, Forest, Genentech, Melinta, Millennium, Pfizer, and Shire; N. L. has contributed to projects for AbbVie, Astellas, BMS, Celgene, Forest, and Sanofi; Y. Z. has contributed for AbbVie, Astellas, Biogen Idec, BMS, Forest, Gilead, Millennium, Shire, UCB, and Vertex; Z. Z. has contributed to projects for AbbVie, Astellas, Bayer, BMS, Genentech, GSK, Shire, Sunovion, and Takeda; J. E. S. has contributed to projects for AbbVie, Boehringer Ingelheim, BMS, Forest, GE Healthcare, Gilead, Janssen, Pfizer, Sunovion, Teva, UCB, Ultragenyx, and Vertex; and E. Q. W. has contributed to projects for AbbVie, Alcon, Astellas, BMS, Ethicon, Forest, Genentech, Millennium, Melinta, Sanofi, Shire, and Vertex.
